# Biotechnological approaches in glucosinolate production

**DOI:** 10.1111/jipb.12705

**Published:** 2018-10-01

**Authors:** Annette Petersen, Cuiwei Wang, Christoph Crocoll, Barbara Ann Halkier

**Affiliations:** ^1^ DynaMo Center Copenhagen Plant Science Centre Department of Plant and Environmental Sciences University of Copenhagen Thorvaldsensvej 40 1871 Frederiksberg C Denmark

## Abstract

Glucosinolates (GLSs) are sulfur‐rich, amino acid‐derived defense compounds characteristic of the Brassicales order. In the past, GLSs were mostly known as anti‐nutritional factors in fodder, biopesticides in agriculture, and flavors in condiments such as mustard. However, in recent times, GLSs have received increased attention as promoters of human health. This has spurred intensive research towards generating rich sources of health‐promoting GLSs. We provide a comprehensive overview of the biotechnological approaches applied to reach this goal. This includes optimization of GLS production and composition in native, GLS‐producing plants, including hairy root and cell cultures thereof, as well as synthetic biology approaches in heterologous hosts, such as tobacco and the microbial organisms *Escherichia coli* and *Saccharomyces cerevisiae*. The progress using these different approaches is discussed.

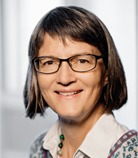

**Barbara Ann Halkier**

**Edited by:** Uwe Sonnewald, Friedrich‐Alexander University, Germany

## INTRODUCTION

### WHY GLUCOSINOLATES?

#### Glucosinolates' role *in planta*

Glucosinolates (GLSs) are important defense compounds present in the Brassicales order, including the brassicaceous vegetables, for example, cabbages, radishes and broccoli (Halkier and Gershenzon 2006). GLSs are hydrolyzed by specific myrosinase enzymes (thio‐β‐glucosidases). The hydrolysis results in unstable aglycones that form − dependent on the type of GLSs, local environment, and presence of specifier proteins − primarily isothiocyanates (ITCs), nitriles, and thiocyanates (Borek et al. 1994; Burow and Wittstock 2009). These hydrolysis products are deterrent or toxic to attackers. GLSs and myrosinases are stored in separate cellular compartments, and only upon tissue disruption, for example by a chewing insect, will they come into contact with each other and hydrolysis occurs (Andréasson et al. 2001).

#### GLSs and health

GLSs, or rather their hydrolysis products, especially the ITCs, have been associated with the health beneficial effects of eating brassicaceous vegetables. More than 40 years ago, Wattenberg (1977) fed aromatic ITCs to rats and observed an inhibition of tumor formation. Ever since, a multitude of studies have linked GLS hydrolysis products to different health beneficial effects. Particularly, 4‐methylsulfinylbutyl GLS (4MSB) and its ITC have received a lot of attention. ITCs have been shown to lower the risk of myocardial infarction (Cornelis et al. 2007) and several kinds of cancer (London et al. 2000; Ambrosone et al. 2004; Kirsh et al. 2007; Zhao et al. 2007; Steinbrecher et al. 2009; Bosetti et al. 2012; Yuan et al. 2016), in addition to having anti‐inflammatory and anti‐microbial properties (reviewed in Saladino et al. 2017). A diet rich in broccoli or broccoli sprouts also showed reduction in LDL cholesterol and oxidative stress markers, both related to increased risk of cardiovascular diseases and cancer (Murashima et al. 2004; Armah et al. 2015).

Conflicting literature exists concerning the effects of ITCs on type‐2‐diabetes. Studies showed both a lowered (Kurotani et al. 2013) and increased (Ma et al. 2018) risk of developing type‐2‐diabetes after enriching participants' diets with brassicaceous vegetables. GLSs and/or ITCs may not help to prevent diabetes, but they can improve insulin resistance in type‐2‐diabetes patients (Bahadoran et al. 2012). At the cellular level, ITCs increase phase II and decrease phase I enzyme activities, regulate oxidative stress, and induce cell cycle arrest and apoptosis, while inhibiting neovascularization (reviewed in Wu et al. 2009; Traka 2016).

Unfortunately, not all GLS hydrolysis products are beneficial. Problems with using brassicaceous vegetables as primary feed for livestock were discovered early on. In 1928, Chesney and coworkers observed how rabbits eating cabbage exhibited swollen thyroid glands, resembling goiter. Cabbage is rich in β‐hydroxyalkenyl GLSs, which upon hydrolysis gives rise to oxazolidine‐2‐thiones. The goitrogenic symptoms observed in the rabbits were attributed specifically to these GLS hydrolysis products, which can hinder iodine uptake by the thyroid (Felker et al. 2016). In other studies, induction of goiter, reduced fertility, and growth inhibition were observed in pigs, poultry, and rodents and to a lesser extent in ruminants and fish (for an extensive review see Tripathi and Mishra 2007).

Severe effects have also been reported in humans. A Chinese woman went into myxedema coma after daily consumption of 1.0–1.5 kg of pak choi over several months (Chu and Seltzer 2010). Myxedema coma is a life‐threatening complication from hypothyroidism, which has a mortality rate of 20%–25% (Klubo‐Gwiezdzinska and Wartofsky 2012). In a later study, participants, who were given kale juice daily for one week, showed increased levels of ITCs in both blood and urine, while uptake of iodine by the thyroid was decreased (Kim et al. 2017). Thus, it is recommended to watch out for iodine malnutrition in individuals who eat large amounts of brassicaceous vegetables. Generally, the literature shows that although some ITCs can be harmful, brassicaceous vegetables are more advantageous than dangerous if consumed in reasonable amounts.

#### GLSs and agriculture

Many important crops exist within the Brassicaceae family, for example, oilseed rape (Gupta 2016). Around the 17^th^ century, the characteristic effects of GLSs and their hydrolysis products were reported, and ever since researchers have tried to understand the underlying mechanisms (Fahey et al. 2001). Fortunately for plant scientists, the Brassicaceae family contains the model plant *Arabidopsis thaliana* and most of the information we have today on biosynthesis, regulation, and function of GLSs is based on studies with this model plant (Koornneef and Meinke 2010).

For agricultural purposes, GLSs are a double‐edged sword. On one hand, they constitute an important defense line for the host plant (Hopkins et al. 2009) and inhibit growth of weeds in surrounding areas (Brown and Morra 1997; Macías et al. 2007). On the other hand, GLSs attract specialized insects (Hopkins et al. 2009) and some non‐brassicaceous crops show growth inhibition from their GLS‐containing neighbors (Brown and Morra 1997; Macías et al. 2007).

The goitrogenic effect upon intake of high GLS content makes the otherwise protein‐rich seed cake of oilseed crops unsuited as animal feed (Marangos and Hill 1975; Hannoufa et al. 2014). Early GLS research focused on reducing GLSs from specific tissues, or the whole plant, because of the growth‐inhibitory effect on other crops, the anti‐nutritional effects, and partially also due to the characteristic bitter taste (Bell et al. 2018). The perhaps most famous example is the 00‐variant of rapeseed (*Brassica napus* L.), from which oil‐rich seeds are used for oil production.

Originally, this plant was unsuited for food and feed production due to its content of erucic acid and GLSs (Schnug and Haneklaus 2016). Variants with low erucic acid content were identified in the early 1960s (Downey and Harvey 1963; Downey and Craig 1964) and named the 0‐variant. These could be used for food oil production; however, the growth‐inhibitory GLSs with their strong taste still made the seedcake unsuited for feed. A decade later, researchers had identified the Bronowski variety with low erucic acid and decreased GLS content (Kondra and Stefansson 1970; Canola Council Canada 2016). This new variant became known as the 00‐variant and today is still the genetic background for the rapeseed cultivars used in agriculture (Schnug and Haneklaus 2016).

After realizing the beneficial effects for plants and humans, the focus shifted to increasing GLS production as is evident from the literature. From the 1960s, many studies concentrated on the allelopathic properties of brassicaceous plants and on how to find varieties with low GLS content. In the early 1980s, this changed into studies of anti‐microbial effects in mostly soil and selection for varieties with high GLS content. For more information on early GLS research, see the review by Brown and Morra (1997).

In the 1990s, “biofumigation” as a GLS‐related agricultural term emerged. The term covers pest control obtained by mulching brassicaceous crops into soil (Kirkegaard and Sarwar 1998). Several studies reported that biofumigation decreased the occurrence of weeds between crops (Borek et al. 1995; Martinez et al. 2006; Rice et al. 2007; Kruger et al. 2016) and reduced attacks from particularly soil‐borne pathogens (Motisi et al. 2009; Clarkson et al. 2015). Unfortunately, biofumigation effects did not always correlate with the GLS content. The explanation was found in the efficiency by which GLSs were broken down (Kirkegaard and Sarwar 1998; Kirkegaard et al. 2000; Morra and Kirkegaard 2002; Gimsing and Kirkegaard 2009). Later studies showed that not all breakdown products were equally efficient against pathogens and that soil environment was crucial in controlling the conversion of GLSs to ITCs with the most efficient conversion rate being 60% (Gimsing and Kirkegaard 2009). Biofumigation is still in use today and as GLSs are considered completely biodegradable, it represents a safe alternative to pesticide use (Badenes‐Perez and Shelton 2006).

As knowledge about the health beneficial effects of GLSs keeps expanding (Traka 2016), it becomes ever more desirable to increase the intake of GLSs. Towards achieving high‐level sources of GLSs multiple approaches have been applied, including classical breeding, transgenic approaches, hairy root and plant cell cultures, as well as microbial production with synthetic biology approaches. Previous reviews on this topic focused on individual biotechnological approaches. In this review, we include all approaches and compare the production levels achieved.

## GLS BIOSYNTHETIC PATHWAYS

More than 130 different GLS structures have been reported (Fahey et al. 2001; Agerbirk and Olsen 2012). The GLSs (and their abbreviations) mentioned in this review are listed in Table 1. GLSs are classified into three groups according to their precursor amino acid: aliphatic GLSs (derived from alanine, isoleucine, leucine, methionine, and valine), aromatic GLSs (derived from phenylalanine and tyrosine) and indolic GLSs (derived from tryptophan) (Halkier and Gershenzon 2006). All GLSs share a common core structure with a glucose moiety connected via an *S*‐glycosidic bond to the C‐atom in a sulfated oxime. The GLS core structure is linked to a side chain derived from the precursor amino acid (Fahey et al. 2001; Agerbirk and Olsen 2012).

**Table 1:** Glucosinolates covered in this review including common names and abbreviations

Name	Abbreviation	Trivial name	Classification
3‐methylthiopropyl GLS	3MTP	Glucoiberverinc	Aliphatic
3‐methylsulfinylpropyl GLS	3MSP	Glucoiberin	Aliphatic
4‐methylthiobutyl GLS	4MTP	Glucoerucin	Aliphatic
4‐methylsulfinylbutyl GLS	4MSB	Glucoraphanin	Aliphatic
4‐hydroxybutyl GLS	4OHB		Aliphatic
3‐butenyl GLS	3BUT	Gluconapin	Aliphatic
*R*‐2‐hydroxy‐3‐butenyl GLS	R‐2OH‐3But	Progoitrin	Aliphatic
*S*‐2‐hydroxy‐3‐butenyl GLS	S‐2OH‐3But	Epiprogoitrin	Aliphatic
indolyl‐3‐methyl GLS	I3M	Glucobrassicin	Indolic
1‐hydroxy‐indolyl‐3‐methyl GLS	1OH‐I3M	1‐Hydroxyglucobrassicin	Indolic
4‐hydroxy‐indolyl‐3‐methyl GLS	4OH‐I3M	4‐Hydroxyglucobrassicin	Indolic
N‐methoxy‐indolyl‐3‐methyl GLS	NMO‐I3M	Neoglucobrassicin	Indolic
4‐methoxy‐indolyl‐3‐methyl GLS	4MO‐I3M	4‐Methoxyglucobrassicin	Indolic
benzyl GLS	BGLS	Glucotropeaolin	Aromatic
2‐phenylethyl GLS	2PE	Gluconasturtiin	Aromatic
*S*‐2‐hydroxy‐2‐phenylethyl GLS	S‐2OH‐2PE	Glucobarbarin	Aromatic
*R*‐2‐hydroxy‐2‐phenylethyl GLS	R‐2OH‐2PE	Epiglucobarbarin	Aromatic
*R*‐2‐hydroxy‐2‐(4‐hydroxyphenyl)ethyl GLS	R‐2OH‐2‐4OHPE	4‐Hydroxyepiglucobarbarin	Aromatic
*p*‐hydroxybenzyl GLS	pOHB	(Gluco)sinalbin	Aromatic

**Figure 1:**
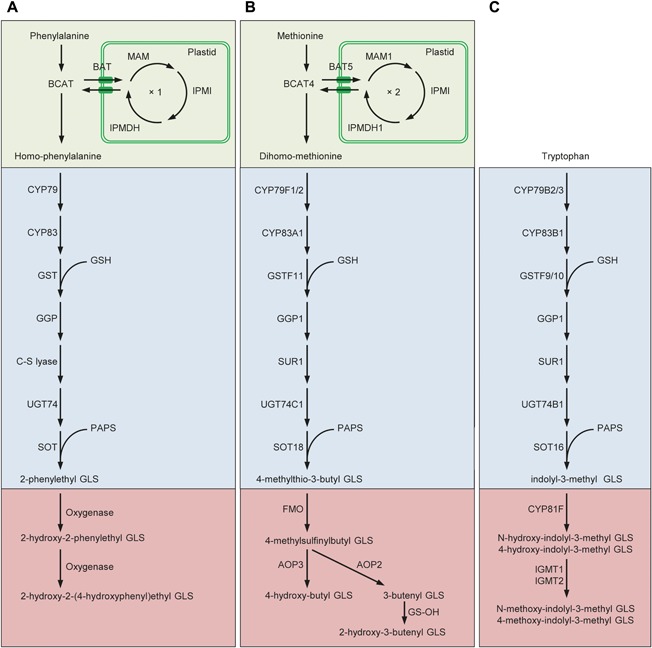
**Examples of biosynthetic pathways of different classes of GLSs** (**A)** Biosynthetic pathway for chain‐elongated, aromatic GLSs (genes unknown). (**B**) Pathway for chain‐elongated, aliphatic GLSs. (**C**) Pathway for indolic GLSs. In the upper (green) part the chain elongation pathway is depicted that is partially plastidic except for the cytosolic BCAT. The middle (blue) part depicts the core structure pathway and the lower (red) part depicts secondary modifications. Abbreviations: GSH, glutathione; PAPS, 3’‐phosphoadenosine‐5’‐phosphosulfate. For gene names, see text.

A comprehensive review on the elucidation of the GLS biosynthetic pathways and genes involved is provided by Sønderby et al. (2010). Briefly, GLSs are synthesized through three processes: chain elongation of selected precursor amino acids (only methionine and phenylalanine), formation of the GLS core structure, and secondary modifications of the amino acid side chain (Figure 1). The chain elongation pathway is comprised of five enzymatic steps, starting with a deamination by a branched‐chain amino acid aminotransferase (BCAT) that converts methionine (or phenylalanine) to a 2‐oxo acid. The 2‐oxo acid then enters a cycle of three successive transformations: condensation with acetyl‐CoA by a methylthioalkylmalate synthase (MAM), isomerization by an isopropylmalate isomerase (IPMI), and oxidative decarboxylation by an isopropylmalate dehydrogenase (IPMDH). The product of these three reactions is a 2‐oxo acid elongated by a single methylene group (–CH_2_–). Subsequently, the molecule can either be transaminated by a BCAT and enter the GLS core structure pathway or proceed through another round of chain elongation (only methionine).

In the core structure pathway, comprised of seven enzymatic steps, precursor amino acids are converted to aldoximes by cytochromes P450 of the CYP79 family. Next, aldoximes are oxidized by cytochromes P450 of the CYP83 family to reactive nitrile oxides that are conjugated with glutathione by glutathione‐*S*‐transferases (GSTs). Cleavage by γ‐glutamate peptidases, GGPs, forms *S*‐alkyl‐thiohydroximates, which are subsequently cleaved by *C‐S* lyases to produce thiohydroximates that are *S*‐glucosylated by glucosyltransferases, UGTs of the 74 family, to form desulfoglucosinolates. Finally, desulfoglucosinolates are sulfated by sulfotransferases, SOTs, to generate GLSs (Figure 1) (Agerbirk and Olsen 2012).

Side chain modifications of aliphatic GLSs are comprised of oxygenations, hydroxylations, alkenylations, and benzoylations (Figure 1B). Flavin monooxygenases are responsible for *S*‐oxygenation to sulfinyl GLSs (Hansen et al. 2007) that are either converted by AOP2 dioxygenases to sulfinyl GLSs, to alkenyl GLSs, or by AOP3 dioxygenases to hydroxyalkyl GLSs (Kliebenstein et al. 2001b). GS‐OH dioxygenases hydroxylate alkenyl GLSs to 2‐hydroxy‐3‐butenyl GLSs. In *A. thaliana*, CHY1, AAO4 and BZO1 are likely responsible for benzoylation of hydroxylated aliphatic GLSs (Kliebenstein et al. 2007; Ibdah and Pichersky 2009; Ibdah et al. 2009) (Figure 1B). For indolic GLSs, hydroxylations are catalyzed by cytochromes P450 of the CYP81F subfamily (Pfalz et al. 2009), followed by methylations by methyltransferases, IGMT1 and IGMT2 in *A. thaliana* (Pfalz et al. 2011) (Figure 1C). For the chain‐elongated, aromatic GLS 2‐phenylethyl GLS (2PE), several unknown oxygenases modify the side chain (Liu et al. 2016b) (Figure 1A).

## ENGINEERING OF GLSs IN BRASSICACEOUS PLANTS

### In planta

Classical breeding has been applied to generate a commercial broccoli with increased levels of 4MSB marketed as a superbroccoli called Beneforté™ (Faulkner et al. 1998). A commercial variant of broccoli (*Brassica oleracea* var. *italica*) was crossed with a wild variant (*B. villosa*) that naturally has higher levels of 4MSB, and a 10‐fold increase in total GLS content in the F1 generation was obtained. Surprisingly, the extracts of the new variant showed a 100‐fold increase in *in vitro* assays detecting induction of phase II detoxification enzymes in cell cultures. The exceptionally high induction was due to a more efficient conversion of GLSs to ITCs as opposed to other breakdown products (Mithen et al. 2003).

Analysis of genomic regions of the wild variant present in the genome of the new variant showed that the master switch for regulating aliphatic GLS, the transcription factor MYB28, was upregulated (Mithen et al. 2003; Traka et al. 2013). Thus, a GMO approach with overexpression of MYB28 as transgene could be applied to obtain a broccoli with increased 4MSB. The vast field of GLS research has resulted in two commercial market products, the 00‐variant of rapeseed and Beneforté™ broccoli.

In 2001, crossings were used to modify the aliphatic GLS composition by eliminating the anti‐nutritional *R*‐2‐hydroxy‐3‐butenyl GLS (R‐2OH‐3But) and upregulating the health‐promoting 4MSB in three *B. oleracea* crops; broccoli, cauliflower, and collard greens (Li et al. 2001). They investigated the effect of four genes, which in *A. thaliana* were shown to regulate the side chain elongation (GLS‐ELONG and GLS‐PRO [de Quiros et al. 2000; Benderoth et al. 2006]), hydroxylation (GLS‐OH, Kliebenstein et al. 2001a), and secondary modification (GLS‐ALK, Kliebenstein et al. 2001b). R‐2OH‐3But is formed from 4MSB through desaturation by GLS‐ALK into 3‐butenyl GLS (3But), which is subsequently hydroxylated by GLS‐OH to R‐2OH‐3But (Li et al. 2001). By introgressing non‐functional variants of GLS‐OH and/or GLS‐ALK alleles the undesired R‐2OH‐3But was eliminated and instead its precursor 4MSB accumulated. These results inspired the use of RNAi to knockdown the GLS‐ALK locus in *B. napus* (Liu et al. 2012). In the best transgenic line, the authors obtained over 60% reduction in R‐2OH‐3But levels and approximately 40 µmol/g 4MSB in the seeds.

Another approach to boost GLS production is by increasing the availability of precursor amino acids. Increased phenylalanine levels in *A. thaliana*, by introducing phenylalanine biosynthesis genes from *E. coli*, resulted in higher levels of benzyl GLS (BGLS) and its ITC (Tzin et al. 2009, 2012). Similar results were obtained by *A. thaliana* mutants overproducing certain amino acids: More phenylalanine lead to more BGLS (Huang et al. 2010) and more methionine lead to more aliphatic GLSs (Inaba et al. 1994).

The CYP79 enzymes are the substrate‐specific entry point to the core structure pathway of which the remaining enzymes are less specific towards the side chain of the precursor amino acids. Accordingly, novel and specific GLS profiles can be engineered by introducing CYP79 genes in a transgenic approach. Brader et al. (2006) introduced CYP79A2 (from *A. thaliana*), CYP79A1 (from *Sorghum bicolor*), and CYP79D2 (from cassava *Manihot esculenta*) into *A. thaliana* to improve plant defense. Expression of CYP79D2 resulted in the accumulation of isopropyl and methylpropyl GLSs with an enhanced resistance against the bacterial soft rot pathogen *Erwinia carotovora*. Expression of CYP79A1, or overexpression of the endogenous CYP79A2, resulted in the accumulation of *p*‐hydroxybenzyl GLS (pOHB) and BGLS, respectively, with increased resistance against the bacterial pathogen *Pseudomonas syringae*. Surprisingly, increased accumulation of the aromatic GLSs showed enhanced susceptibility to the fungus *Alternaria brassicicola* (Brader et al. 2006). The latter shows that the outcome of an engineering strategy can be difficult to predict.

Interestingly, overexpression of *AOP2* from *B. oleracea* in *A. thaliana* resulted in a 2‐fold increase of total aliphatic GLS content, suggesting a push‐pull effect (Wentzell et al. 2007). Introduction of *AOP2* transcripts increased transcript levels for genes in the entire aliphatic biosynthetic pathway. The precursor, methylsulfinylalkyl GLSs, was efficiently converted into the corresponding alkenyl GLSs upon overexpression of *AOP2* (Neal et al. 2010). Whether or how sensing of the individual GLSs occurs to change the flux through the pathway is currently unknown.

As a less biotechnological, but potentially very efficient approach, researchers have exploited that the availability of sulfur can directly boost the production of GLSs. The special sulfur chemistry of GLSs is due to the presence of at least two sulfur atoms in each GLS molecule: in the sulfate group originating from 3’‐phosphoadenosine‐5’‐phosphosulfate (PAPS) and in the thio‐glucose moiety with the S originating from cysteine in glutathione. Methionine‐derived GLSs may have a third sulfur atom in their structures dependent of the side chain modifications. Falk et al. (2007) reviewed how sulfur feeding to different plant species under various cultivation conditions increased GLS production. As an example, BGLS levels were increased more than 50‐fold after feeding with sulfate (Matallana et al. 2006).

In summary, classical breeding as well as transgenic approaches have been successful in modifying GLS content in brassicaceous plants for increasing both health benefits and resistance to pathogen attacks. Noticeably, simple sulfate feeding yielded the highest increase in GLSs. The approaches differ in their ability to enable global increase in GLSs, or enrichment of a specific GLS, so the choice of strategy will depend on the purpose, for example, pest resistance or nutritional value.

### Cell cultures

Plant cell cultures are a popular choice for production of specialized metabolites. They have the advantage of being plant tissue cultivated in liquid cultures. Similar to microbial cultures, plant cell cultures allow for several optimization strategies, such as precursor feeding and media and cultivation modifications. In addition, strain improvement through screening or engineering as well as elicitors have been successfully used to increase production (Bhatia and Bera 2015). Several attempts have been made at producing GLSs in cell cultures. These are summarized in Table 2, and selected examples will be described in the following section.

**Table 2:** Overview of different approaches to produce glucosinolates in native and heterologous hosts

Classification	Tissue	Approach/treatment	Type of GLS	Highest yield	References
**Brassicaceous plants**					
*A. thaliana* Col‐0	Rosette (7‐week‐old)	No treatment	Total GLS	24.23 μmol/g DW	Kastell et al. 2013a
	Root (7‐week‐old)	No treatment	Total GLS	8.02 μmol/g DW	
	Cell culture	No treatment	Total GLS	2.59 μmol/g DW	
	Hairy root culture	No treatment	Total GLS	5.35 μmol/g DW	
*A. thaliana* Col‐0 (transgenic lines)	Rosette (7‐week‐old)	35S::*AtCYP79F1* or	Total GLS	∼ 25 μmol/g DW	Kastell et al. 2015
	Root (7‐week‐old)	35S::*AtCYP79F2* or	Total GLS	∼ 27 μmol/g DW	
	Hairy root culture	35S::*BrCYP79F1*	Total GLS	∼ 10 μmol/g DW	
*A. thaliana* Col‐0 (transgenic lines)	Cell culture	35S::*MYB28*	Total GLS	∼ 2.3 μmol/g FW	Hirai et al. 2007
*A. thaliana* Col‐0	Hypocotyl (9‐d‐old)	No treatment	Total GLS	0.7 μmol/g FW	Alvarez et al. 2008
	Hypocotyl (9‐d‐old)	50 μM MeJA	Total GLS	1 μmol/g FW	
	Cell culture	No treatment	Total GLS	1.4 μmol/g FW	
	Cell culture	50 μM MeJA	Total GLS	4 μmol/g FW	
Chinese cabbage (*B. rapa* ssp. *pekinenses*) (transgenic lines)	Hairy root culture	35S::*AtMAM1*	Total GLS	∼ 2 μmol/g FW	Zang et al. 2008a
	Hairy root culture	35S::*AtCYP79F1* / 35S::*AtCYP83A1*	Total GLS	∼ 2.5 μmol/g FW	
Chinese cabbage (*B. rapa* ssp. *pekinenses*) (transgenic lines)	Leaf	35S::*AtCYP79B3* / 35S::*AtCYP83B1*	Total GLS	∼ 3.5 μmol/g FW	Zang et al. 2008b
	Hairy root culture	35S::*AtCYP79B3* / 35S::*AtCYP83B1*	Total GLS	∼ 1.6 μmol/g FW	Zang et al. 2009
Chinese cabbage (*B. rapa* ssp. *pekinenses*)	Leaf	0.2 mmol/L MeJA + 2 mmol/L SA	Total GLS	92.08 μmol/g DW	Zang et al. 2015
	Root	0.2 mmol/L MeJA + 2 mmol/L SA	Total GLS	241 μmol/g DW	
Broccoli (*B. oleracea* var. *italica*)	Hairy root culture	No treatment	Total GLS	17.86 a.u.	Kim et al. 2013
	Hairy root culture	0.1 mg/L NAA	Total GLS	20.02 a.u.	
	Hairy root culture	0.1 mg/L IBA	Total GLS	23.00 a.u.	
	Hairy root culture	0.1 mg/L IAA	Total GLS	28.02 a.u.	
Kale (*B. oleracea* var. *acephala*)	Hairy root culture	Full B5 media	Total GLS	22.24 μmol/g DW	Lee et al. 2016
Kale (*B. oleracea* var. *acephala*)	Leaf	0.5 mmol/L sulfate feed	Total GLS	10.59 μmol/g DW	Park et al. 2018
	Leaf	2 mmol/L sulfate feed	Total GLS	26.8 μmol/g DW	
Indian cress (*T. majus)*	Callus	No treatment	BGLS	∼ 34 μmol/g DW	Wielanek and Urbanek 1999
	Cell culture	No treatment	BGLS	∼ 44 μmol/g DW	
	Hairy root culture	No treatment	BGLS	∼ 85 μmol/g DW	
Indian cress (*T. majus)*	Hairy root culture	0.6 mmol/L Phe + 0.6 mmol/L Cys + 0.2 mmol/L ASA	BGLS	85.8 μmol/g FW	Wielanek and Urbanek 2006
Water cress (*N. officinale)*	Hairy root culture	No treatment	2PE + BGLS	31.33 μmol/g DW	Wielanek et al. 2009
	Hairy root culture	0.5 mmol/L Phe + 0.5 mmol/L Cys	2PE + BGLS	142 μmol/g DW	
Land cress (*B. verna*)	Hairy root culture	No treatment	2PE	95.9 μmol/g DW	
	Hairy root culture	0.5 mmol/L Phe + 0.5 mmol/L Cys	2PE	236 μmol/g DW	
Mountain rock cress (*A. caucasica*)	Hairy root culture	No treatment	3MTP	32.3 μmol/g DW	
	Hairy root culture	0.5 mmol/L Met + 0.5 mmol/L OAS	3MTP	197 μmol/g DW	
Water cress (*N. officinale)*	Hairy root culture		Total GLS	0.34 μmol/g DW	Park et al. 2011
Yellowcress (*N. montanum*)	Callus	200 ppm Trp	IAN	∼ 0.04 μmol/g FW	Songsak and Lockwood 2004
	Cell cultures	200 ppm Trp	IAN	∼ 0.12 μmol/g FW	
Celandine spider flower (*C. chelidonii*)	Callus	200 ppm Met + 200 ppm Cys	Methyl ITC	∼ 0.003 μmol/g FW	
	Cell cultures	200 ppm Met + 200 ppm Cys	Methyl ITC	∼ 0.04 μmol/g FW	
White mustard (*S. alba*)	Leaf	No treatment	Total GLS	∼ 60 μmol/g DW	Kastell et al. 2013b
	Root	No treatment	Total GLS	∼ 15 μmol/g DW	
	Hairy root culture	No treatment	Total GLS	∼ 10 μmol/g DW	
	Hairy root culture	100 μmol JA	Total GLS	∼ 20 μmol/g DW	
Turnip (*B. rapa* ssp. *rapa*)	Leaf	No treatment	Total GLS	∼ 9 μmol/g DW	
	Root	No treatment	Total GLS	∼ 20 μmol/g DW	
	Hairy root culture	No treatment	Total GLS	∼ 10 μmol/g DW	
	Hairy root culture	50 μmol JA	Total GLS	∼ 80 μmol/g DW	
**Non‐brassicaceous plants**					
*Nicotiana benthamiana*	Leaf (12 dpi)	Transient expression	BGLS	0.57 μmol/g FW	Geu‐Flores et al. 2009
*Nicotiana benthamiana*	Leaf (6 dpi)	Transient expression	BGLS	∼ 1.8 μmol/g FW	Møldrup et al. 2011
*Nicotiana tabacum*	Leaf (13‐week‐old)	Transgenic lines	BGLS	0.5 μmol/g FW	Møldrup et al. 2012
*Nicotiana benthamiana*	Leaf	Transient expression	Indolic GLSs	No quantification	Pfalz et al. 2011
*Nicotiana benthamiana*	Leaf (7 dpi)	Transient expression	4MSB	0.04 μmol/g FW	Mikkelsen et al. 2010
**Microorganisms**					
*Saccharomyces cerevisiae*	Culture (32 hai)	Genomic integration	I3M	1.07 mg/L	Mikkelsen et al. 2012
*Escherichia coli*	Culture	Plasmid	4MSB	No quantification	Yang et al. 2018

dpi, days post infiltration; DW, dry weight; FW, fresh weight; hai, hours after inoculation; IAN, indoleacetonitrile; JA, jasmonic acid.

As information on GLSs is primarily obtained from *A. thaliana*, this plant has naturally been studied for GLS production in cell cultures. *A. thaliana* Col‐0 cell culture produced only 0.26 μmol/g dry weight (DW). Overexpression of the MYB28 transcription factor, a key regulator of aliphatic GLSs, in *A. thaliana* cell culture resulted in an increase to approximately 2.3 μmol/g fresh weight (FW), which is still roughly 1.5‐fold lower than in the rosette of a wild‐type plant (Hirai et al. 2007). These results indicate that it is possible to increase aliphatic GLSs in cell cultures without simultaneously increasing, for example, indolic GLSs, but much optimization is needed to match even the production levels of an intact plant.

Induction of GLS production in cell cultures by treatment with 50 μM methyl jasmonate (MeJA) for 24 h increased the GLS production with a higher induction of indolic GLSs compared to aliphatic GLSs (Alvarez et al. 2008). The total GLS content increased from 1.4 μmol/g FW to approximately 4 μmol/g FW in cell cultures and, thereby, surpassed the previously reported titers. *A. thaliana* appears not to be the best GLS producer in cell cultures. Instead, various cress species were studied as production hosts for GLSs; the advantage being that the cress species often have a simple GLS composition. Cell cultures of yellow cress (*Nasturtium montanum*) produced 18 μg/g FW aromatic GLSs (Wielanek and Urbanek 1999), which is 4.5‐fold higher than reported from *A. thaliana*. However, these levels are much lower than what was reported from cell cultures of Indian cress (*Tropaeolum majus*), which produced 44 μmol/g DW BGLS (Wielanek and Urbanek 1999).

In summary, cress species appear superior to *A. thaliana* as host for GLS production in cell cultures. By far the highest production reported in cell cultures was seen in Indian cress after substrate feeding. The relatively limited studies of GLS production in plant cell cultures include media optimization and elicitation, as well as the use of different species. None of these matches the levels of GLSs present natively in the intact plant. Based on this observation, plant cell cultures appear to be an unsuitable approach for GLS production.

### Hairy root cultures

A given plant tissue can be converted into excessive root tissue by infection with *Agrobacterium rhizogenes*. Such hairy root cultures have been reported to induce specialized metabolism (Bulgakov 2008) and, in some cases, secrete the produced compounds (Fukui et al. 1999; Medina‐Bolivar et al. 2007; Tatsumi et al. 2016). The latter provides a cost‐efficient extraction process and potentially boosts production by preventing feedback inhibition. In Table 2, GLS production in hairy root cultures reported to date is summarized, of which selected examples will be discussed below.

Several groups have reported GLS production in hairy root cultures made from various brassicaceous vegetables. *A. thaliana* is a popular host plant for hairy root production of GLSs, due to a comprehensive mutant library and several reported GLS overproducers. A comparison of GLS content in different tissues of *A. thaliana* Col‐0 hairy root cultures showed that the total aliphatic GLS content was significantly lower in hairy roots (1.27 μmol/g DW) than in leaf tissue (18.69 μmol/g DW) and roots (4.70 μmol/g DW) (Kastell et al. 2013a). Also, the hairy root culture produced more indolic GLSs than the roots of the plant, but still less than seen in the leaf tissue. The total GLS content in leaves and roots was 24.23 μmol/g DW and 8.02 μmol/g DW, respectively, whereas the hairy roots produced 5.35 μmol/g DW.

In 2015, the same group attempted to engineer higher aliphatic GLS production by overexpressing the *CYP79F1* or *CYP79F2* genes that are entry points in the aliphatic core structure pathway (Kastell et al. 2015). In the transgenic plants (T3 generation), aliphatic GLSs were up by 1.5‐ to 3.5‐fold, and indolic GLSs were increased by 1.5‐ to 2.0‐fold. In the corresponding hairy root cultures, the overall GLS content was lower despite an increase in *CYP79F1* and *CYP79F2* transcripts (Kastell et al. 2015), which is in accordance with the previous report (Kastell et al. 2013a). This down‐regulation of specialized metabolism contradicts previous reports on production in hairy roots (Bulgakov et al. 2013), but it fits with all reports concerning specifically GLS production. Hairy root cultures often have an increased ratio of indolic GLSs to aliphatic GLSs, which could be explained by a similar pattern in root tissue of the plant (Kastell et al. 2015).

Hairy root cultures for GLS production have also been reported for white mustard (*Sinapis alba*) and turnip (*B. rapa* ssp. *rapa*) (Kastell et al. 2013b). A comparison of the levels in hairy root cultures with that of the leaf and normal root tissues showed that, in turnip, the leaf tissue and hairy root culture had similar GLS levels (approximately 9–10 μmol/g DW), whereas the roots produced about 20 μmol/g DW. In white mustard, the leaves had the highest GLS content at 60 μmol/g DW, whereas the hairy root culture and roots contain approximately 10–15 μmol/g DW. Treatment with elicitors showed that the highest GLS content was measured in white mustard 14 days after treatment with 100 μmol jasmonic acid (JA) (approximately 20 μmol/g DW), whereas in turnip GLS levels increased to approximately 80 μmol/g DW 14 days after treatment with 50 μmol JA. These yields are better than most of the reported values, but they do not compare to what was seen with elicitors and substrate feeding in cress species (see below). Hairy root cultures of kale (*B. oleracea* var. *acephala*) contained only four indolic GLSs: indolyl‐3‐methyl GLS (I3M), 4‐hydroxy‐I3M (4OH‐I3M), 4‐methoxy‐I3M (4MO‐I3M) and N‐methoxy‐I3M (NMO‐I3M) at a total GLS concentration of 22.24 μmol/g DW when grown in full B5 media (Lee et al. 2016). Elicitation did not improve GLS production in this case.

As for the cell cultures (see above), several cress species have been tested as a GLS source in hairy roots. With the BGLS‐producing Indian cress (*T. majus*), hairy roots produced approximately 85 μmol/g DW (Wielanek and Urbanek 1999). Treatment of the hairy root culture with cysteine increased BGLS content by 150%, whereas phenylalanine, peptone and MeJA induced GLS formation by 30%–50%; however, both cysteine and phenylalanine inhibited biomass production after 6 days (Wielanek and Urbanek 1999). In a later report, this group also tried optimization of BGLS production by feeding with a phenylalanine analogue, (L)‐(1‐amino‐2‐phenylethyl) phosphonic acid. Noticeable, when all three compounds (cysteine, phenylalanine, and (L)‐(1‐amino‐2‐phenylethyl) phosphonic acid) were added the production increased by 415% compared to the control and without inhibiting biomass production (Wielanek and Urbanek 2006). The highest production was 2,245 mg/100 mL culture (approximately 76 μmol/g FW). Further addition of hormone elicitors showed that the best response was in acetylsalicylic acid‐treated cultures, where the BGLS content increased 3‐fold to 1,698 mg/100 mL culture (50.5 μmol/g FW). Acetylsalicylic acid, in combination with phenylalanine and cysteine feeding, resulted in BGLS content to a maximum of 2,497 mg/100 mL culture (85.8 μmol/g FW) (Wielanek and Urbanek 2006).

Wielanek et al. (2009) investigated aromatic and aliphatic GLSs in hairy root cultures from watercress (*Nasturtium officinale*) and land cress (*Barbarea verna*) that both have particularly high levels of 2PE, and from mountain rock cress (*Arabis caucasica*) that produces aliphatic GLSs (Barillari et al. 2001; Bennett et al. 2004; Jeon et al. 2017). The hairy root cultures showed great variation, but looking at the best line of each species land cress produced high amounts of 2PE (95.9 μmol/g DW), mountain rock cress produced only 3‐methylthiopropyl (3MTP) GLS (79.5 μmol/g DW), and watercress produced both BGLS and 2PE (74.6 μmol/g DW) (Wielanek et al. 2009). Subsequently, elicitors and precursors were fed to boost production further. Phenylalanine, cysteine, methionine, serine and O‐acetyl‐L‐serine (OAS) were added individually, or in combinations, always in identical concentrations (0.5 mmol/L).

For watercress, all elicitors and supplements except serine increased production of GLSs (Wielanek et al. 2009). For watercress and land cress the highest production was seen with the combination of phenylalanine and cysteine. Approximately 142 μmol/g DW GLS was produced in watercress and 236 μmol/g DW in land cress. In mountain rock cress, the highest GLS production was seen with a combination of methionine and OAS (approximately 197 μmol/g DW). These results are higher than any other reports on GLSs in hairy root cultures and are particularly promising for production as only one GLS in land cress and mountain rock cress and two GLSs in watercress were measured.

However, other GLSs may be present although not described in this study. Another study on hairy root cultures of watercress established that at least two indolic GLSs are present in addition to the aromatic 2PE and BGLS (Park et al. 2011). This study also reported significantly lower levels of GLSs than seen in previous work (Wielanek et al. 2009). The different hairy root lines ranged from 0.14–0.34 μmol/g DW, and interestingly only 57% were aromatic GLSs (Park et al. 2011). This could indicate that indolic GLS production, which is usually low in the plant, is upregulated when watercress is transformed into a hairy root culture, as seen in most other reports.

In summary, several optimization strategies have been used for developing hairy root cultures for GLS production, including media composition, substrate feeding, elicitation, genetic modification, and mechanical wounding. Varying outcomes are reported dependent on species and treatment. However, the cultures all seem to share an induction of indolic GLSs compared to the corresponding plant. For the most part, the total GLS content was also lower in the cultures and the composition was quite distinct. Unfortunately, the yields reported here do not compare with GLS levels in the intact plants and must be considered insufficient for large‐scale production.

## ENGINEERING OF GLSs IN NON‐BRASSICACEOUS PLANTS

In the last decade, much research on establishment of GLS biosynthetic pathways in the non‐brassicaceous tobacco plant was reported. The first example of *de novo* synthesis of GLSs in non‐brassicaceous plants was the engineering of BGLS in *Nicotiana benthamiana* (Geu‐Flores et al. 2009) as a fast approach to test the feasibility of engineering projects (Voinnet et al. 2003). BGLS was produced when five *A. thaliana* genes (*CYP79A2*, *CYP83B1*, *SUR1*, *UGT74B1*, and *SOT16*) were transiently expressed in *N. benthamiana*. Two metabolic bottlenecks related to the addition of reduced and oxidized sulfur, respectively, were identified.

First, accumulation of a GSH conjugate of the product of CYP83B1 resulted in the discovery of the missing enzyme γ‐glutamyl peptidase 1, GGP1 (Geu‐Flores et al. 2009). Co‐expression of GGP1 eliminated the GSH conjugate accumulation, increased BGLS content and identified another bottleneck at the sulfotransferase step (Møldrup et al. 2012). Sulfation of desulfobenzyl GLS, the last intermediate in the pathway, is catalyzed by the sulfotransferase SOT16 and requires PAPS as co‐substrate. Addition of *A. thaliana* APK2 kinase, active in the PAPS generation cycle, resulted in efficient conversion of phenylalanine to BGLS without accumulation of intermediates. When BGLS production was stably engineered into *Nicotiana tabacum* (Møldrup et al. 2012), *Plutella xylostella* (diamondback moth) − a specialist that uses GLSs as oviposition stimuli − laid its eggs on the BGLS‐producing tobacco, and the eggs did not hatch (unpubl. results). This provided proof‐of‐concept for genetically modified dead‐end trap crops.

The pathway for indolic GLSs was engineered into *N. benthamiana* to investigate the role of CYP81F subfamily in secondary modifications of the indole ring (Pfalz et al. 2011). When the four members of the CYP81F subfamily (*CYP81F1*, *CYP81F2*, *CYP81F3* and *CYP81F4*) were co‐infiltrated with indolic GLS biosynthetic genes, I3M was converted to 4OH‐I3M by CYP81F1, CYP81F2, and CYP81F3, but not CYP81F4, and all four CYP81Fs catalyzed I3M to 1OH‐I3M. However, GLS profiles of individual *cyp81f* mutants in *A. thaliana* showed that NMO‐I3M levels were reduced substantially in the *cyp81f4* mutant without the other CYP81Fs being able to compensate, which suggests that CYP81F4 is mainly responsible for hydroxylating at the C1‐position.

4MSB was produced in *N. benthamiana*, demonstrating the feasibility of engineering chain‐elongated GLSs (Mikkelsen et al. 2010). The 4MSB pathway consists of a four‐gene chain elongation pathway, a seven‐gene core structure pathway, and a flavin‐monooxygenase (FMO) responsible for the final *S*‐oxygenation step. The biosynthetic pathway is compartmentalized with the chain elongation enzymes in the plastid, except for the cytosolic BCAT4, and the core structure pathway and FMO are located in the cytosol.

Transient expression of the chain elongation genes in tobacco leaves resulted in accumulation of homo‐methionine and dihomo‐methionine, the products of one and two cycles of chain elongation, as well as the corresponding by‐products homo‐(iso)leucine and dihomo‐(iso)leucine (Mikkelsen et al. 2010). The latter may reflect that the methionine chain elongation pathway has evolved from leucine biosynthesis; i.e., chain elongation of valine to leucine (Halkier and Gershenzon 2006).

Dihomo‐methionine accumulation was increased by more than 50‐fold when BCAT4 was targeted to the chloroplast, suggesting that production of the methionine‐derived α‐keto acid in the same compartment as the remaining chain elongation machinery is beneficial. Co‐expression of BCAT3 had no measurable effect on the metabolites produced, indicating that the final transamination reaction was catalyzed by an endogenous tobacco activity, or possibly BCAT4, although *in vitro* data indicate that BCAT4 does not catalyze this transamination reaction (Schuster et al. 2006).

When genes of the entire pathway were expressed 3MTP, 3‐methylsulfinylpropyl (3MSP), 4‐methylthiobutyl (4MTB), and 4MSB GLS were produced, as well as the chain‐elongated leucine‐ or isoleucine‐derived GLSs not found in the native *A. thaliana* (Mikkelsen et al. 2010). The addition of GSTF11 increased 4MSB production by 20%. Remarkably, no dihomo‐methionine was detected when the genes of the entire pathway were co‐expressed. This result suggests that the dihomo‐methionine biosynthesis is rate‐limiting and the plastid‐produced chain‐elongated amino acids have been transferred to the cytosol, where they are accessible to the ER‐anchored cytoplasmic CYP79F1.

Later, optimization of the dihomo‐methionine production in *N. benthamiana* resulted in a 9‐fold increase, to approx. 430 nmol/g FW, using a different combination of genes for methionine chain elongation (Crocoll et al. 2016) compared to the previously reported results (Mikkelsen et al. 2010). Co‐expression of the large subunit (LSU1) and small subunit (SSU3) of IPMI from *A. thaliana* resulted in a 21‐fold increase of dihomo‐methionine production, instead of only expressing IPMI‐SSU3 as previously reported (Mikkelsen et al. 2010). Co‐expression of the transporter protein BAT5 and a cytosolically localized BCAT4 resulted in higher dihomo‐methionine levels than in combination with only chloroplast‐targeted BCAT4, suggesting that BAT5 is an efficient antiporter for the chain‐elongated α‐keto acids. Co‐expression of IPMDH3 resulted in higher dihomo‐methionine levels than with IPMDH1, although another study suggested that IPMDH1 was the key player in methionine chain elongation (He et al. 2013).

In summary, the transgenic approach in tobacco successfully produced GLSs, albeit the levels were low compared to level in the brassicaceous plants. The advantage of a heterologous host is production of only the desired GLS as opposed to a mixture of GLSs. However, introduction of the compartmentalized methionine chain elongation pathway resulted in a mixture of chain‐elongated GLSs, also from other amino acids. The stable transgenic line produced a mere 0.5 μmol/g FW BGLS (Møldrup et al. 2012), while in comparison the highest levels of BGLS in transiently expressing plants were 1.8 μmol/g FW BGLS (Møldrup et al. 2011). As is also evident from studies with cell cultures and hairy root cultures, the aliphatic GLSs are produced at much lower levels than indolic and aromatic GLSs as exemplified with 0.04 4MSB μmol/g FW transiently produced in tobacco (Mikkelsen et al. 2010).

## ENGINEERING OF GLSs IN MICROBIAL HOSTS

Microbial hosts are suitable for large‐scale production. Today, two microbial hosts have been used for GLS production: *Escherichia coli* and *Saccharomyces cerevisiae*. First, production of the basic indolic GLS, I3M, in *S. cerevisiae* was obtained by stable integration of *A. thaliana* genes from the core indolic GLS pathway into the yeast genome (Mikkelsen et al. 2012). This proved that simple GLSs derived directly from protein amino acids (i.e. without prior chain elongation) can be produced in yeast. Initially, GSTF9 and a P450 reductase, ATR1, were not included. However, production of the GSH conjugate intermediate increased by 2.6‐fold when GSTF9 and ATR1 were added. The final indolic GLS production was 1.07 mg/L.

Three reports have been published related to GLS engineering in *E. coli*. Liu et al. (2016a) reported the production of the benzyl ITC from BGLS by generating multiple *E. coli* strains with four genes (*CYP79A2*, *CYP83B1*, *UGT74B1*, and *SOT18*) of the aromatic core structure pathway from *A. thaliana* along with a myrosinase from the aphid *Brevicoryne brassicae* and MetC from *E. coli* to replace *A. thaliana* C‐S lyase (SUR1). The P450 enzymes were N‐terminally modified as previously described (Wittstock and Halkier 2000) and fused to *A. thaliana* cytochrome P450 reductase ATR2. Extracts from the individual *E. coli* strains were combined in an *in vitro* assay. The production titer of benzyl ITC was not reported.

Towards engineering 4MSB, associated with the health benefits of broccoli, a first step is to make the precursor amino acid dihomo‐methionine. When expressing the *A. thaliana* chain elongation pathway, both homo‐methionine and dihomo‐methionine, as well as the corresponding chain‐elongated leucine derivatives accumulated in the media (Mirza et al. 2016). When methionine was added to the media, levels of homo‐ and dihomo‐methionine increased significantly, while the leucine‐derived products decreased. The production titer was reported to be 57 mg/L dihomo‐methionine with similar levels of homo‐methionine and approximately 20 mg/L leucine‐derived products (Mirza et al. 2016).

Recently, the problem of unwanted byproducts was seemingly circumvented by choosing *BCAT3* and *MAM1* genes from other *Brassica* species, which were supposedly less promiscuous (Yang et al. 2018). The authors expressed all genes of the 4MSB pathway from multiple plasmids and chose genes from *A. thaliana* (*LSU1*, *SSU3*, *IPMDH1*, *UGT74B1*, *SOT18*, and *FMO1*), *B. rapa* (*BCAT3* and *CYP83B1*), *B. oleracea* (*MAM1* and *CYP79F1*) and a fungus *Neurospora crassa* (*SUR1*). *GGP1* and *GST* genes were not included and the P450 enzymes were modified by fusing them together with ATR2. Expression of the 13 genes resulted in detectable production of 4MSB (Yang et al. 2018). No quantification was reported, so the titers cannot be compared to the production of indolic GLSs in *S. cerevisiae*.

Microbial engineering of GLSs is still early research and only two studies have successfully produced GLSs *in vivo* and only one could quantify this production. Generally, the threshold for production levels considered financially sustainable is in the grams‐per‐liter range. By this standard, the GLSs produced in microbial hosts are approximately 1,000‐fold too low for large‐scale production. This problem can appear daunting. However, many strategies for optimization are still open for investigation, for example, multivariate optimization and directed evolution approaches (Yadav et al. 2012; Abatemarco et al. 2013). Current literature established that production can be increased by hundred‐ and even thousand‐fold when combining different optimization strategies (Yoshikuni et al. 2008; Ajikumar et al. 2010; Morrone et al. 2010; Nybo et al. 2017).

As an alternative approach to microbial engineering for the production of GLSs or ITCs for human health, Ho et al. (2018) optimized the conversion of the consumed GLSs into ITCs at the site of cancer *in vivo*. In a mouse study, a normal healthy diet with GLSs from brassicaceous vegetables was supplemented with an *E. coli* strain that produces a myrosinase and is engineered to bind specifically to colon cancer cells. The ITCs release was thereby focused close to a tumor. The authors reported 95% inhibition of cancer cell growth *in vitro* and reduced tumors *in vivo* of mice fed with the engineered *E. coli* and brassicaceous vegetables.

## PERSPECTIVES

As a means to enrich average intake of health‐promoting GLSs, high levels of 4MSB were successfully obtained in the Beneforte™ broccoli using a classical breeding approach. Other approaches have focused on identification of suitable hosts for high GLS production. GLS engineering in heterologous hosts poses several challenges. The special sulfur chemistry associated with GLSs and for which the cruciferous hosts have learnt to cope, gives the native hosts an advantage over other hosts and gives synthetic biology approaches unique challenges. For the chain‐elongated GLSs, which include the health‐promoting 4MSB, the ability to control the number of cycles in the iterative process constitutes an additional challenge.

GLSs production through plant cell or hairy root cultures have improved in some species of brassicaceous plants, particularly cress species. Unfortunately, they are still far from economically sustainable in large‐scale production and − for the most cases − have a complex mixture of different GLSs, which introduces the need for downstream purifications. Hence, engineering microbes represent an appealing alternative. Current reports indicate that tremendous optimizations are needed for this to be a viable option.

In addition to classical optimization strategies such as media composition, growth conditions, and construct designs, other methods for greater production includes directed evolution on production hosts, metabolic flux analysis, as well as protein engineering to modulate enzyme kinetics and specification. These options involve testing production in hundreds − if not thousands − of mutants in high‐throughput screening platforms. This is made feasible only by emerging computational software and robotic laboratory equipment. New biotechnology tools are expected to enable high‐level production of glucosinolates in the near future.
